# Exploring genetic markers of adult obesity risk in black adolescent South Africans—the Birth to Twenty Cohort

**DOI:** 10.1038/nutd.2015.7

**Published:** 2015-06-15

**Authors:** V Pillay, N J Crowther, M Ramsay, G D Smith, S A Norris, Z Lombard

**Affiliations:** 1 Division of Human Genetics, School of Pathology, Faculty of Health Sciences, National Health Laboratory Service & University of the Witwatersrand, Johannesburg, South Africa; 2 Department of Chemical Pathology, School of Pathology, Faculty of Health Sciences, National Health Laboratory Service & University of the Witwatersrand, Johannesburg, South Africa; 3 Sydney Brenner Institute for Molecular Bioscience, Wits Bioinformatics, University of the Witwatersrand, Johannesburg, South Africa; 4 MRC Integrative Epidemiology Unit, University of Bristol, Bristol, UK; 5 MRC/Wits Developmental Pathways for Health Research Unit, Department of Paediatrics, School of Clinical Medicine, Faculty of Health Sciences, University of the Witwatersrand, Johannesburg, South Africa; 6 School of Molecular & Cell Biology, Faculty of Science, University of the Witwatersrand, Johannesburg, South Africa

## Abstract

To date more than 90 loci that show an association with body mass index (BMI) and other obesity-related traits, have been discovered through genome-wide association studies. These findings have been widely replicated, mostly in European and Asian populations, but systematic investigation in African cohorts is still lacking. Therefore, the aim of our study was to replicate the association of six single-nucleotide polymorphisms (SNPs) previously linked to BMI, in a South African black adolescent cohort. The SNPs were in or near *GNPDA2* (rs10938397), *MTCH2* (rs10838738), *NEGR1* (rs2568958), *SH2B1* (rs7498665), *STK33* (rs10769908) and *TMEM18* (rs6548238). The SNPs were genotyped in 990 adolescents from the Birth to Twenty study, using an Illumina VeraCode assay, and association with BMI statistically assesed by using PLINK. Three of the SNPs tested were associated with BMI in this African cohort, and showed a consistent (albeit smaller) directional effect to that observed in non-African cohorts. We identified significant association between BMI and rs10938397 (effect allele-G) near *GNPDA2* (*P*_adj_=0.003), rs7498665 (effect allele-G) in *SH2B1* (*P*_adj_=0.014) and rs6548238 (effect allele-C) near *TMEM18* (*P*_adj_=0.030). This data suggests that common genetic variants potentially contributes to obesity risk in diverse population groups.

## Introduction

Recent studies show that the mean prevalence of overweight and obesity (combined) in South African children and adolescents is ~15%.^1^ The prevention of childhood obesity is a key global health priority as obesity is a major contributor to increased mortality in adulthood,^2^ and is increasing in prevalence in both developed and developing nations, such as South Africa.

The risk of developing obesity is modulated by both heritable and environmental factors.^3^ Heritability studies of body mass index (BMI) demonstrated that a significant proportion of the variance in BMI (40–70%) is because of genetics^4^ with genome-wide association studies of obesity-related traits identifying more than 90 risk loci thus far.^5, 6, 7^ An explicit deficit of African-centric genome-wide association studies data for body composition and obesity exist, especially for sub-Saharan African populations. In addition, most published genetic association studies of BMI have primarily focussed on the association with adult BMI. Identifying loci that predispose to obesity early in life could provide a better understanding of the early determinants of adult obesity and may also uncover potential new targets for the therapeutic prevention of obesity.

Previously, our group investigated the role of gene variants in appetite regulating genes with BMI in an adolescent cohort,^8^ replicating single-nucleotide polymorphism (SNP) associations in *FTO* and *MC4R*, as well as establishing a novel association with variants in the *LEP* gene. Here, we describe the replication within the same cohort of six variants, selected based on previous evidence of robust association with BMI in non-African populations in large meta-analyses undertaken by the GIANT consortium.^9, 10, 11^ These include SNPs in or near *GNPDA2*, *MTCH2*, *NEGR1*, *SH2B1*, *STK33* and *TMEM18*.

## Subjects and methods

We used data and samples from participants of the Birth to Twenty (Bt20) study that have been described in detail elsewhere.^12^ Written assent was obtained from all adolescents in conjunction with written consent from caregivers, prior to a blood sample collection. This study was approved by the Human Research Ethics Committee of the University of the Witwatersrand (certificate number M010556). The participants in the Bt20 cohort are South Africans who self-identified as Sotho speakers (that is, of southeastern Bantu-speaking descent), thus belonging to the Niger-Kordofanian ethno-linguistic group. A subset of individuals (43%, *n*=990) from the Bt20 cohort were randomly selected for this study, and consisted of 524 (53%) female and 466 (47%) male adolescents, with a mean (±s.d.) age of 13.7±0.2 years. Anthropometric measurements were obtained by using standard methods^13^ and pubertal stage was assessed by using a validated self-assessment method.^14^

SNPs previously associated with BMI^9, 10, 11^ were selected for analysis, and include rs2568958 near *NEGR1*, rs6548238 near *TMEM18*, rs10938397 near *GNPDA2*, rs10769908 in *STK33*, rs10838738 in *MTCH2* and rs7498665 in *SH2B1*. Genotyping was performed using the GoldenGate VeraCode assay (Illumina, San Diego, CA, USA). Internal quality control was performed on all raw genotype data according to the supplier's specifications using the genotyping module of BeadStudio (Framework version 3.1.3.0; Illumina). Further quality control filters based on minor allele frequency (MAF<0.01) and Hardy–Weinberg equilibrium (HWE<0.05) were used as exclusion criteria. Ancestry information markers previously genotyped in this cohort show no significant population structure within the cohort.^8^

PLINK v.1.9 software was used for all statistical analysis, unless stated otherwise.^15^ The distribution of BMI was skewed and therefore was log-transformed to normality for all analyses. Linear regression was used to assess the association of the selected SNPs with BMI. As BMI correlated significantly with gender, pubertal stage and age, analyses were adjusted for these variables by including them in the linear model as covariates (*P*_adj_). Given the strong prior information about the correlation of the SNPs tested here with BMI, we considered this a replication study, and therefore *P*-values below 0.05 were considered significant. Given the minor allele frequencies of the SNPs tested, the study achieves 80% power to detect differences among the means of BMI, with a standard F-test for linear regression, as previously calculated.^8^ To assess the combined impact of risk alleles on BMI, we calculated a risk allele score by summing the number of BMI-increasing alleles per individual. This score was calculated including the risk alleles described here, as well as the four previously identified BMI-associated variants in this cohort: *FTO* (rs17817449), *LEP* (rs10954174 and rs6966536) and *MC4R* (rs17782313).^8^

## Results

The study group consisted of 524 (53%) female and 466 (47%) male adolescents, with a mean (±s.d.) age of 13.7±0.2 years. Summary statistics and trends related to BMI in this subset were described previously,^8^ and based on these data all analyses were adjusted for gender, gender-specific pubertal stage and age.

Table 1 reports the association of the SNPs with (log)BMI. Of the six SNPs investigated, associations with BMI were replicated for three SNPs in this African cohort, and showed a similar (albeit smaller) directional effect to that observed in the discovery studies. We identified significant correlations between BMI and rs10938397 (effect allele-G) near *GNPDA2* (*P*_adj_=0.003), rs7498665 (effect allele-G) in *SH2B1* (*P*_adj_=0.014), and with rs6548238 (effect allele-C) near *TMEM18* (*P*_adj_=0.030).

**Figure 1:**
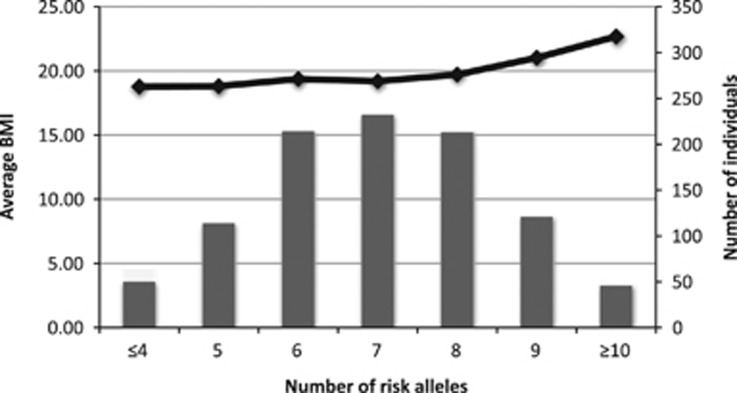
Combined impact of risk alleles on average BMI in the Bt20 cohort. Risk alleles were summed for each individual. The number of individuals in each risk allele category is shown along the *x* axis.

To assess the combined impact of risk alleles on BMI, we calculated a risk alllele score (Figure 1), in which we included the three risk alleles described here, as well as four previously identified in the same cohort.^8^ The difference in average BMI between individuals with a high genetic-susceptibility score (defined as having ⩾10 BMI-increasing alleles) and those with a low genetic-susceptibility score (⩽4 BMI-increasing alleles) was 3.90 kg m^−^^2^, signifying a 21.7% increase in average BMI between these two groups. In comparison, if only the three SNPs from this paper is assesed, the increase in average BMI (comparing the lowest with the highest risk score) is 2.06 log kg m^−^^2^, signifying a 10.5% increase in average BMI between these two groups. This implies that both set of risk SNPs contribute to the effect that we see on BMI.

## Discussion

Several common genetic variants have been robustly associated with adult obesity risk. This study provides confirmation that three of these variants are also associated with BMI in a South African cohort of adolescents. The G-allele of both rs7498665 (*SH2B1)* and rs10938397 (*GNPDA2)*, and the C-allele of rs6548238 (*TMEM18)* were shown to be associated with an increase in BMI. A similar, albeit smaller, directional effect was observed to that seen in the discovery studies in non-African cohorts. These results demonstrate that genetic variants for adult BMI are also associated with BMI earlier in life, which may provide insights into the genetic aetiology of obesity within an indigenous African population.

*GNPDA2*, *TMEM18* and *SH2B1* are all highly plausable biological candidates for adiposity—all three are expressed in the brain, with evidence of a role in appetite regulation or affecting adipose tissue biology.^16^ GNPDA2 is expressed in the hypothalamus, alluding to a neuronal influence on energy balance, and has been associated with BMI in both pediatric^17^ and adult cohorts, including a replication in an African-American cohort.^18^ *SH2B1*'s link to metabolic function is well established^19^ and deletions in this gene are associated with severe early-onset obesity.^20^ Furthermore, rs7498665 in *SH2B1* is a missense variant and results in a substitution of alanine with threonine, which likely affects protein activity and expression. TMEM18 is ubiquitously expressed, and although a direct link to obesity is still elusive, early evidence suggests a likely role through transcriptional regulation of critical targets.^16^

We acknowledge a number of limitations in our study. The sample size is moderate and therefore not powered to detect small effects on BMI, suggesting that the potential contribution of *MTCH2* and *STK33* warrants follow-up investigation in a larger cohort. The significant differences in the genomic structure between Africans and non-Africans genomes, could lead to a situation where SNPs shown to be associated with a trait in European populations may be weak predictors for causal variants in African populations, due to difference in linkage disequilibrium.^21^ Another consideration is that, although BMI is an established obesity index, it is not the best indicator of adiposity^22^ and the use of more suitable measures may help to elucidate the role of genetics in adiposity.

Finally, the data in this study are derived from a cohort of adolescents in the midst of puberty. It is therefore possible that the effects on weight of some polymorphisms may have been masked by puberty-associated changes in body fat mass. Furthermore, the effects of some polymorphisms on BMI are conceivably only observed later in life. Elucidating the genetic component of obesity in children is important because it may uncover factors that have a stronger phenotypic effect than those gene variants that only become apparent after years of exposure to an obesogenic environment. Also, gene variants than give rise to childhood obesity may provide important information on important metabolic or neurological pathways that could be therapeutically manipulated to reduce adipose tissue accumulation. The discovery of such polymorphisms may also help identify individuals with a high risk of obesity and hence allow early lifestyle interventions.

In conclusion, our study has replicated associations for increased BMI with SNPs present in or near *TMEM18*, *SH2B1* and *GNPDA2* in an African adolescent population. These observations suggest that variants in these genes or neighbouring loci may be important in body weight regulation in divergent populations.

## Figures and Tables

**Table 1:** Results of the six SNPs tested for association with log(BMI) in the Bt20 cohort

*Nearest gene*	*SNP*	*Chr*	N	*Alleles*^a^			P-*values*		
				*Effect*^b^	*Other*	*Frequency effect allele*	P_*unadj*_	P_*adj*_^c^	*Effect size* *Beta^d^ (s.e.m)*
*GNPDA2*	rs10938397	4	961	G	A	0.21	0.002	**0.003**	0.013(0.004)
*MTCH2*	rs10838738	11	982	G	A	0.07	0.680	0.739	−0.003(0.007)
*NEGR1*	rs2568958	1	976	A	G	0.44	0.713	0.837	0.001(0.004)
*SH2B1*	rs7498665	16	963	G	A	0.29	0.086	**0.014**	0.007(0.004)
*STK33*	rs10769908	11	985	C	T	0.25	0.663	0.701	−0.002(0.004)
*TMEM18*	rs6548238	2	982	C	T	0.92	0.086	**0.030**	0.011(0.006)

Abbreviations: BMI, body mass index; NCBI, National Center for Biotechnology Information; SNP, single-nucleotide polymorphism. Significant *P*-values are shown in bold.

a Allele coding according to the forward strand (NCBI dbSNP Build 134).

b Effect allele associated with increased BMI in the original study.

c *P*-values are adjusted for age, sex and sex-specific pubertal stage.

d Effect sizes in log kgm^−2^).
